# Enhanced osteogenesis and angiogenesis by mesoporous hydroxyapatite microspheres-derived simvastatin sustained release system for superior bone regeneration

**DOI:** 10.1038/srep44129

**Published:** 2017-03-13

**Authors:** Wei-Lin Yu, Tuan-Wei Sun, Chao Qi, Hua-Kun Zhao, Zhen-Yu Ding, Zhi-Wang Zhang, Ben-Ben Sun, Ji Shen, Feng Chen, Ying-Jie Zhu, Dao-Yun Chen, Yao-Hua He

**Affiliations:** 1 Department of Orthopedics, Shanghai Jiao Tong University Affiliated Sixth People’s Hospital, 600 Yishan Road, Shanghai 200233, China; 2 State Key Laboratory of High Performance Ceramics and Superfine Microstructure, Shanghai Institute of Ceramics, Chinese Academy of Sciences, 1295 Dingxi Road, Shanghai 200050, China; 3 University of Chinese Academy of Sciences, 19 Yuquan Road, Beijing 100049, China; 4 Shanghai Jiao Tong University Affiliated Sixth People’s Hospital, School of Biomedical Engineering, 600 Yishan Road, Shanghai 200233, China.

## Abstract

Biomaterials with both excellent osteogenic and angiogenic activities are desirable to repair massive bone defects. In this study, simvastatin with both osteogenic and angiogenic activities was incorporated into the mesoporous hydroxyapatite microspheres (MHMs) synthesized through a microwave-assisted hydrothermal method using fructose 1,6-bisphosphate trisodium salt (FBP) as an organic phosphorous source. The effects of the simvastatin-loaded MHMs (S-MHMs) on the osteogenic differentiation of rat bone marrow mesenchymal stem cells (rBMSCs) and angiogenesis in EA.hy926 cells were investigated. The results showed that the S-MHMs not only enhanced the expression of osteogenic markers in rBMSCs but also promoted the migration and tube formation of EA.hy926 cells. Furthermore, the S-MHMs were incorporated into collagen matrix to construct a novel S-MHMs/collagen composite scaffold. With the aid of MHMs, the water-insoluble simvastatin was homogenously incorporated into the hydrophilic collagen matrix and presented a sustained release profile. *In vivo* experiments showed that the S-MHMs/collagen scaffolds enhanced the bone regeneration and neovascularization simultaneously. These results demonstrated that the water-insoluble simvastatin could be incorporated into the MHMs and maintained its biological activities, more importantly, the S-MHMs/collagen scaffolds fabricated in this study are of immense potential in bone defect repair by enhancing osteogenesis and angiogenesis simultaneously.

For better bone tissue regeneration, pleiotropic bone substitutes with both osteogenic and angiogenic activities are desirable for the management of bone defects caused by trauma, tumors, infections or genetic malformations. There is a close connection between osteogenesis and angiogenesis during the process of bone repair, and a temporal and coordinated angiogenic response is of great importance for successful bone repair^1^. Neovascularization within the bone defects not only provides nutrients, oxygen, calcium and phosphate but also transports mesenchymal stem cells to facilitate bone regeneration^2^. Therefore, the development of synthetic biomaterials coupling osteogenesis and angiogenesis has attracted increasing attention in recent years.

Statins, inhibitors of the competitive 3-hydroxy-3-methyl coenzyme A (HMG-CoA) reductase, have been widely used to treat hyperlipidemia and hypercholesterolemia. It has been demonstrated that statins, including simvastatin, can induce osteoblastic differentiation of bone marrow mesenchymal stem cells and improve the osteogenesis of human or animal osteoblastic cell lines^3^,^4^,^5^,^6^,^7^,^8^. Moreover, statins have been demonstrated to enhance angiogenesis through up-regulation of gene expression of vascular endothelial growth factor (VEGF) and basic fibroblast growth factor (FGF-2)^6^,^9^,^10^. Both VEGF and FGF-2 have been shown to induce BMP-2 expression and stimulate osteoblast differentiation indirectly^11^,^12^. Cui *et al*. reported that locally applied simvastatin promoted critical-sized calvarial defects healing by recruitment of autogenous osteogenic stem cells and endothelial progenitor cells^13^. It seems likely that a single administration of simvastatin may enhance new bone formation by the two different pathways of osteogenesis and angiogenesis. However, little attention has been paid to promoting bone regeneration by coupling osteogenic and angiogenic effects of simvastatin together.

Despite the advantages of statins in bone regeneration, systemic administration of statins with clinical doses required for lipid-lowering therapy would not be able to produce desirable effects on bone anabolism and catabolism^14^. However, high doses of statins may result in increasing chances of statin-related adverse effects such as rhabdomyolysis and inflammatory myopathies^15^. Alternatively, similar or superior bone anabolic effects can be expected from lower and sustainable release of statins by a local delivery method. Various biomaterials, including poly(L-lactic acid) (PLA), poly(lactic-co-glycolic acid) (PLGA), calcium sulfate, hyaluronic acid, gelatin and methylcellulose have been investigated for the local administration of simvastatin with slow-release drug delivery^13^,^16^,^17^,^18^,^19^,^20^,^21^. However, there are disadvantages to synthetic hydrophobic carriers such as PLA or PLGA, which may induce stronger inflammatory responses in the body and take longer to complete *in vivo* degradation compared with hydrogel carriers of drug release^22^,^23^,^24^. Moreover, it is practically impossible to achieve the homogenous distribution and sustainable release of water-insoluble simvastatin in hydrophilic carriers such as hyaluronic acid, gelatin, and methylcellulose. Therefore, development of biomaterials with better biocompatibility and controllable biodegradability remains a challenge for the sustained delivery of water-insoluble simvastatin.

Recently, nanostructured calcium phosphate (CAP) porous microspheres have attracted increasing attention because of their high specific surface area, hierarchical architecture, good biocompatibility and bioactivity^25^,^26^,^27^. Nanostructured CAP porous microspheres are promising drug-carriers because of their high specific surface area, excellent biocompatibility and biodegradability. For instance, gentamicin-loaded hydroxyapatite porous microspheres exhibited a sustained drug release profile, and vancomycin could be loaded efficiently on the surface of nano-hydroxyapatite particles and showed an obvious and continuous antimicrobial performance^28^,^29^. In recent years, we have reported the microwave-assisted rapid synthesis of CAP porous microspheres using biomolecules such as creatine phosphate and adenosine 5′-triphosphate disodium salt (ATP) as organic phosphorus sources, and these nanostructured porous microspheres had high drug loading capacities and showed immense potential in the application of drug delivery^30^,^31^,^32^. Moreover, it was reported that the addition of nano-hydroxyapatite to a collagen/alginate composite contributed to the increase in both mechanical and biological properties^33^. Therefore, nanostructured CAP porous microspheres are potential candidates for the delivery of simvastatin and may encourage bone regeneration when combined with collagen matrix that has been optimized for bone tissue engineering.

Based on our previous work, the MHMs were synthesized through a microwave-assisted hydrothermal method using FBP as the phosphorus source, and the MHMs were used as the direct carrier of simvastatin. The osteogenic and angiogenic activities of the S-MHMs were investigated systematically *in vitro*. Furthermore, a novel S-MHMs/collagen composite scaffold was developed through a lyophilization fabrication process. With the aid of MHMs, water-insoluble simvastatin was homogenously incorporated into the hydrophilic collagen matrix. Then, the capacities of the S-MHMs/collagen scaffold to stimulate bone regeneration and neovascularization *in vivo* were assessed in a rat critical-sized calvarial defect model by micro-computed tomography (micro-CT) measurements and histological assays.

## Results

### Characteristics of the MHMs

**Figure 1:**
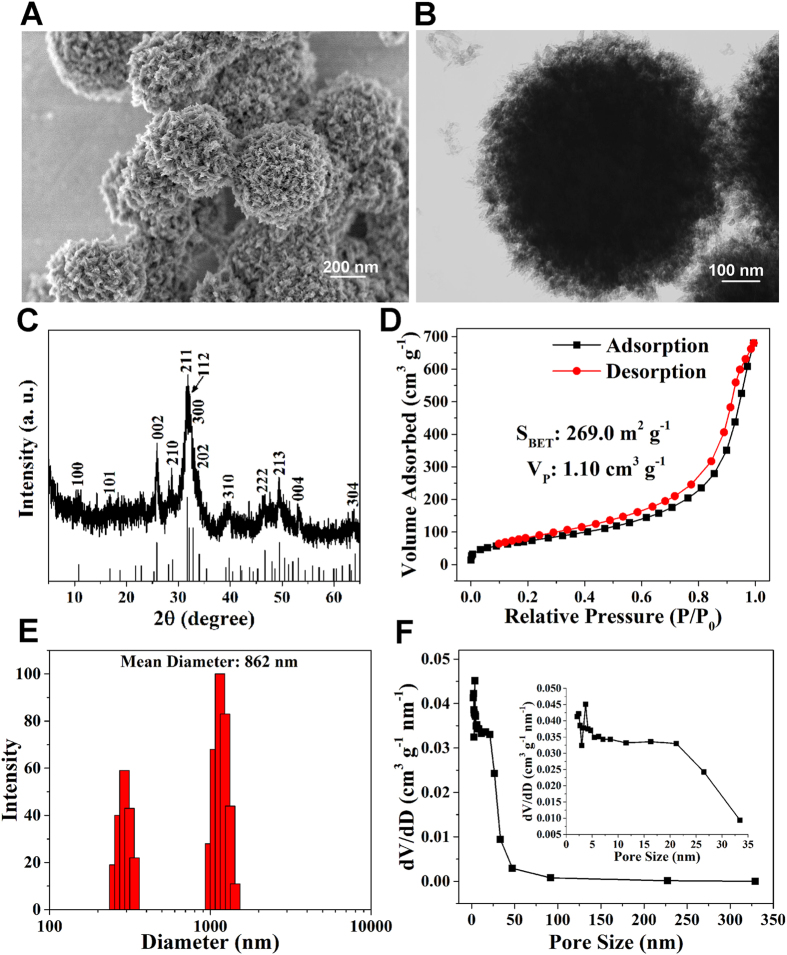
Physical characteristics of MHMs. (**A**) SEM image; (**B**) TEM image; (**C**) XRD pattern; (**D**) Nitrogen adsorption-desorption isotherm curve; (**E**) DLS size distribution; (**F**) BJH pore size distribution curve.

As shown in Fig. 1A and B, the MHMs were consisted of hydroxyapatite nanosheets/nanorods that were hierarchically assembled into nanostructured mesoporous microspheres. Figure 1C shows the XRD pattern of the MHMs, which was indexed to a single phase of hydroxyapatite with a hexagonal structure (Ca_10_(PO_4_)_6_(OH)_2_, JCPDS No. 09–0432). Figure 1D shows that the BET specific surface area and the Barrett−Joyner−Halenda (BJH) desorption cumulative volume (*V*_P_) of the MHMs were 269.0 m^2^ g^−1^ and 1.1 cm^3^ g^−1^, respectively. The average size of MHMs detected by dynamic light scattering (DLS) was 862 nm (Fig. 1E), which was slightly larger than the ~600 nm estimated by microscopy. Figure 1F shows that the MHMs possessed macro- and mesoporous structures, and the average BJH desorption pore size was 11.3 nm.

### Simvastatin loading and release behavior

**Figure 2:**
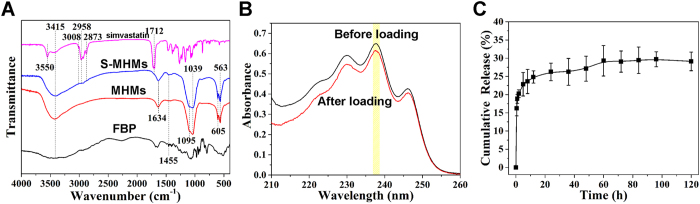
Simvastatin loading and release assay. (**A**) FTIR spectra of FBP, MHMs S-MHMs and simvastatin; (**B**) UV-vis absorption spectra of the ethanol solution of simvastatin before and after loading; (**C**) Simvastatin release curve in NS solution of S-MHMs.

As shown in Fig. 2A, the adsorption of simvastatin on the MHMs was supported by FTIR spectroscopy. The absorption peaks at 3008, 2958 and 2873 cm^−1^ (C–H stretching vibrations), and 1712 cm^−1^ (stretching vibration of ester and lactone carbonyl functional groups) were assigned to simvastatin^34^. Before and after simvastatin loading, the concentrations of simvastatin in the ethanol solution were measured by UV-vis absorption spectroscopy at a wavelength of 238 nm to calculate the simvastatin loading capacity of MHMs (Fig. 2B). The simvastatin loading capacity of the MHMs was 75.7 mg g^−1^, and the loading efficiency was 7.6%. As shown in Fig. 2C, after immersion in normal saline for 2 h, the S-MHMs showed a burst release about 20.23 ± 1.08%, and the release behavior of S-MHMs trended toward an equilibrium release (29.27 ± 4.17%) for 60 h.

### Effects of MHMs and S-MHMs on cell viability and ALP activity

**Figure 3:**
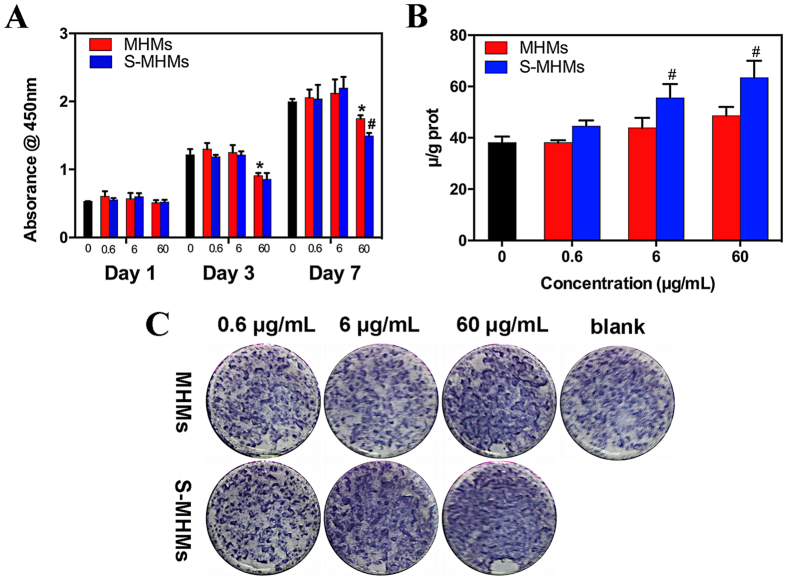
The effects of MHMs and S-MHMs on cell viability and ALP activity. (**A**) Proliferation of rBMSCs; (**B**) Quantification of ALP activity; (**C**) ALP staining. (*Comparison between the MHMs group and the blank control, ^#^comparison between MHNs and S-MHMs groups at the same concentration, *p* < 0.05).

The CCK-8 assay was performed to evaluate the proliferation of rBMSCs cultured with different concentrations of MHMs and S-MHMs (Fig. 3A). Both the MHMs and S-MHMs showed no cytotoxicity at 0.6 and 6 μg/mL. However, the cell proliferation rate dropped when the concentrations of MHMs and S-MHMs reached 60 μg/mL. It is notable that S-MHMs at 60 μg/mL showed stronger cytotoxicity than the MHMs. The effects of the S-MHMs on the proliferation of EA.hy926 cells are shown in Supplementary Figure S1.

The ALP activity of rBMSCs cultured with different concentrations of MHMs and S-MHMs was examined on day 10. According to the quantitative analysis, the ALP activity of the rBMSCs cultured with 6 and 60 μg/mL of S-MHMs was higher than that of the rBMSCs cultured with MHMs or medium alone (Fig. 3B). Moreover, the ALP staining of rBMSCs cultured with S-MHMs was more intensive than that of the rBMSCs cultured with MHMs or medium alone on day 10 (Fig. 3C).

### Expression of osteogenesis-related genes of rBMSCs cultured with MHMs and S-MHMs

**Figure 4:**
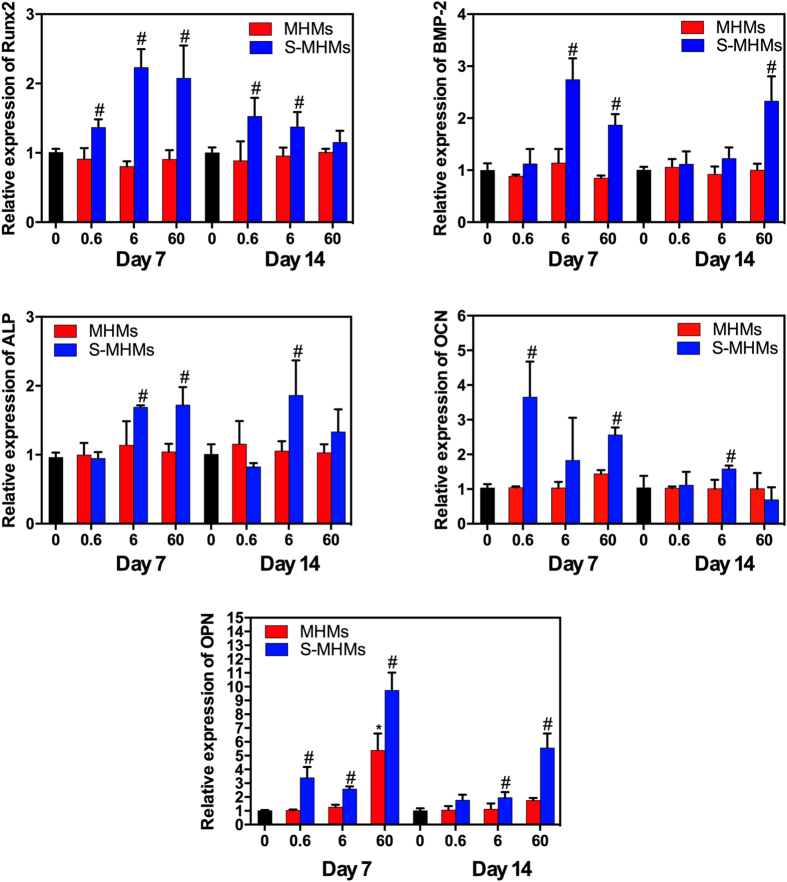
The effects of S-MHMs on the expression of osteogenesis-related genes in rBMSCs on days 7 and 14. (*Comparison between the MHMs group and the blank control, #comparison between MHMs and S-MHMs groups at the same concentration, *p* < 0.05).

The RT-qPCR results showed that the S-MHMs stimulated the osteogenic differentiation of rBMSCs (Fig. 4). Enhanced expression of Runx2 was observed for the cells cultured with S-MHMs on day 7, and significant differences were observed for the cells cultured with 0.6 and 6 μg/mL of S-MHMs on day 14. The rBMSCs showed remarkably increased expression of BMP-2 on day 7 when cultured with 6 and 60 μg/mL of S-MHMs, and significant difference was observed only when the cells cultured with 60 μg/mL of S-MHMs on day 14. The ALP gene expression was significantly improved for the cells cultured with 6 and 60 μg/mL of S-MHMs on day 7, and the expression of ALP was significantly upregulated when the cells cultured with 6 μg/mL of S-MHMs on day 14. The expression of OCN was elevated for the cells cultured with different concentrations of S-MHMs on day 7, whereas significant difference was observed only when the cells cultured with 6 μg/mL of S-MHMs on day 14. The rBMSCs cultured with S-MHMs showed higher expression of OPN compared with those cultured with MHMs on days 7 and 14.

### Cell migration and tube formation assays

**Figure 5:**
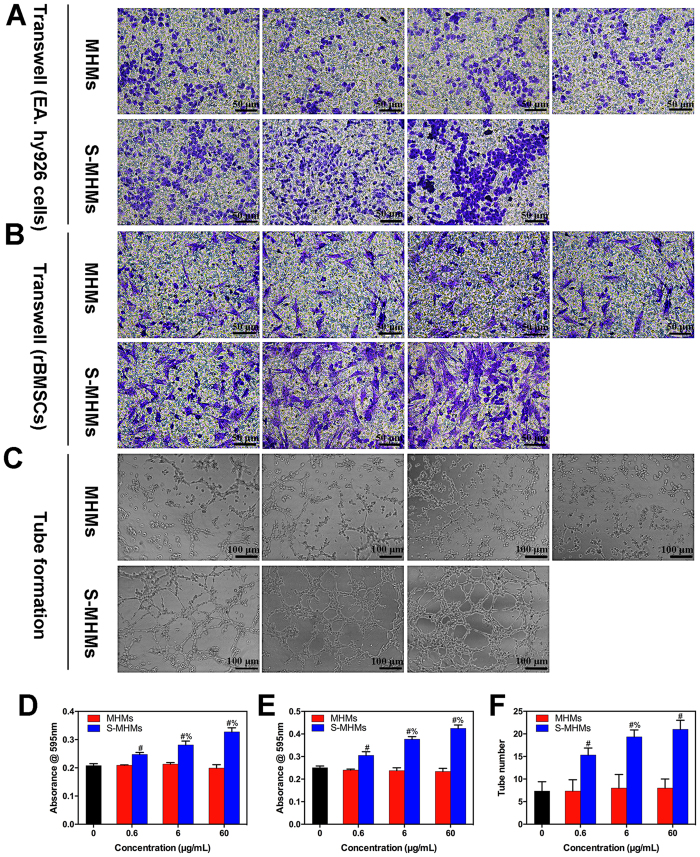
Representative photographs showing the effects of S-MHMs on the migration of EA.hy926 cells (**A**,**D**) and rBMSCs (**B**,**E**), and the tube formation of EA.hy926 cells (**C**,**F**). (^#^Comparison between the MHMs and S-MHMs at the same concentration, ^%^comparison between 0.6 and 6 μg/mL of S-MHMs, 6 and 60 μg/mL of S-MHM, *p* < 0.05).

Transwell migration assay showed that the S-MHMs promoted the migration of EA.hy926 cells and rBMSCs in a concentration-dependent manner compared with the MHMs (Fig. 5A,B,D,E). Assay of the tube formation was used to evaluate the pro-angiogenic potential of the S-MHMs. After 8 hours of incubation on the Matrigel, the S-MHMs enhanced the tube formation of EA.hy926 cells in a concentration-dependent manner compared with the MHMs (Fig. 5C,F). The EA.hy926 cells pretreated with S-MHMs showed elongated and tubule-like structures, whereas the cells pretreated with MHMs or medium alone exhibited an incomplete or sparse tubular network.

### Effects of S-MHMs on HIF-1α and VEGF expression in rBMSCs

**Figure 6:**
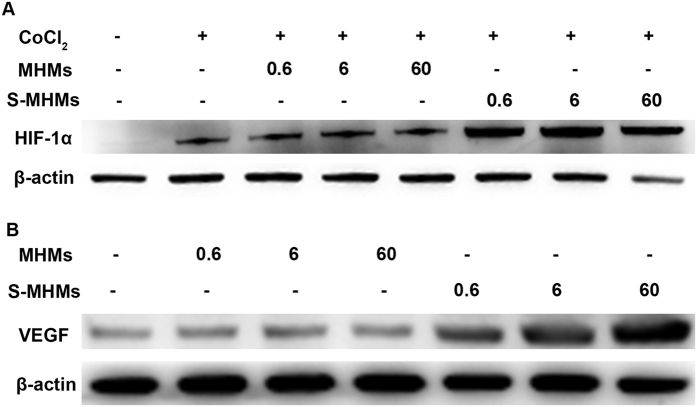
Western blot analysis for the expression of HIF-1α (**A**) and VEGF (**B**) in rBMSCs cultured with different concentrations of MHMs and S-MHMs for 48 h.

The expression of HIF-1α and VEGF in rBMSCs was detected by western blot. The results showed that the rBMSCs cultured with MHMs at different concentrations or medium alone showed low levels of expression of HIF-1α and VEGF. In contrast, the S-MHMs enhanced the expression of HIF-1α and VEGF in a concentration-dependent manner (Fig. 6).

### Scaffolds characterization and simvastatin release

**Figure 7:**
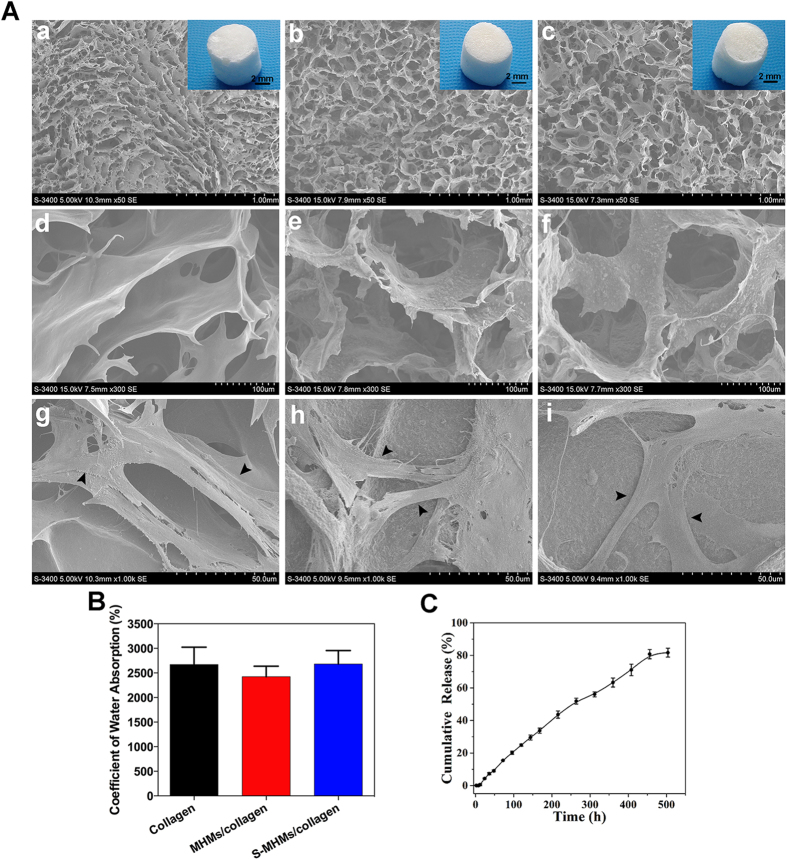
Scaffolds characterization and simvastatin release. (**A**) SEM images of the collagen, MHMs/collagen and S-MHMs/collagen scaffolds and cell attachment (arrowheads) on the scaffolds; (**B**) Water absorbing capacities of the scaffolds; (**C**) Simvastatin release from the S-MHMs/collagen scaffold.

As shown in Fig. 7A–a,b and c, the collagen, MHMs/collagen and S-MHMs/collagen scaffolds were highly porous and exhibited a hierarchical structure. The pore structure was uniformly distributed and highly interconnected with an average size of approximate 200 μm. Higher magnification images show that the surface of the collagen scaffold was smooth (Fig. 7A–d), whereas the surfaces of the MHMs/collagen and S-MHMs/collagen scaffolds were quite rough (Fig. 7A–e,f), indicating that the MHMs and S-MHMs were uniformly incorporated within the collagen matrix. Figure 7A–g, h and i show that the rBMSCs attached to the surface of pore struts and spread well in a decent morphology on each type of scaffold.

The water absorbing capacities of the scaffolds are shown in Fig. 7B. The hydroscopicity of the collagen, MHMs/collagen and S-MHMs/collagen scaffolds was 2672 ± 352%, 2427 ± 211% and 2682 ± 274%, respectively, indicating these scaffolds were highly porous with similar porosity.

Figure 7C shows the simvastatin release profile of the S-MHMs/collagen scaffold. The S-MHMs/collagen scaffold showed a sustained release profile, and the release behavior of simvastatin trended toward an equilibrium release period for 504 h (21 days). The cumulative released simvastatin from the scaffold was approximately 81.69%. No burst release of simvastatin was observed.

### Cellular viability and morphology on the scaffolds

**Figure 8:**
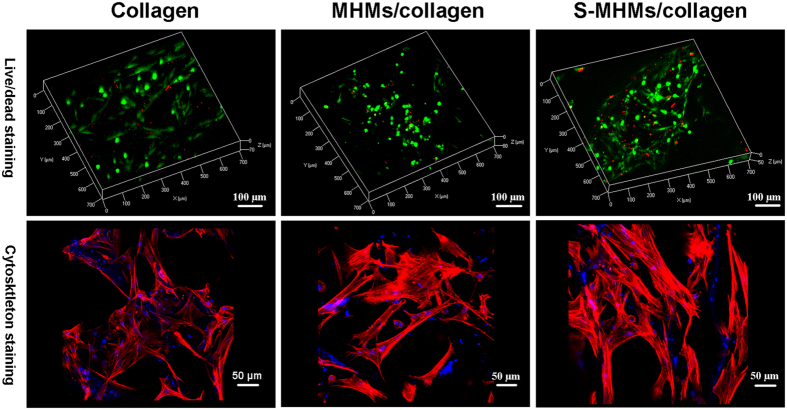
The live/dead staining and cytoskeleton staining of rBMSCs seeded on the scaffolds after culturing for 3 days. (The viable cells were stained green whereas the dead cells were stained red; The cytoskeleton was stained red whereas the cell nuclei were stained blue).

After culturing for 3 days, the live/dead staining demonstrated that most of the seeded cells stayed alive, and only a small number of dead cells were observed (Fig. 8). The cytoskeleton staining showed that cells on all the three types of scaffolds presented a well-spread morphology (Fig. 8), which is consistent with the SEM observation.

### Micro-CT analysis

**Figure 9:**
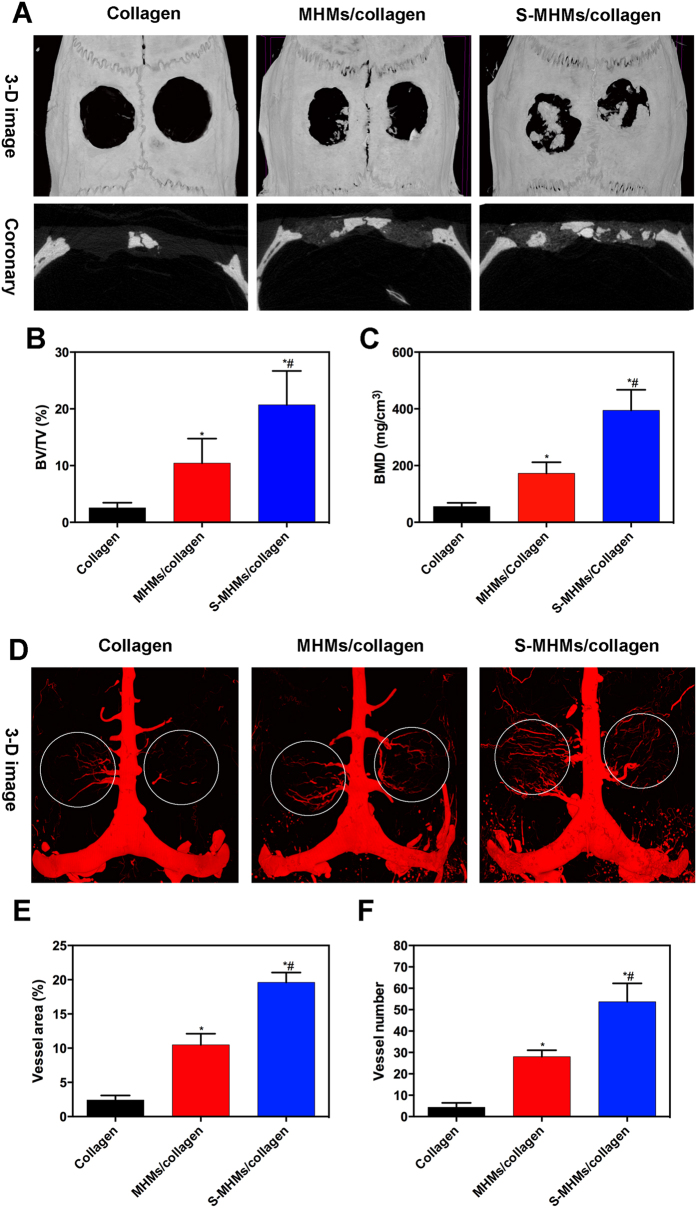
New bone formation and neovascularization evaluated by micro-CT. (**A**) 3-D and coronary views of reconstructed calvaria; (**B**,**C**) BMD and BV/TV in the defects; (**D**) New blood vessels presented by 3-D reconstruction images; (**E**,**F**) Quantitative analysis of the new blood vessel area and number. (*Comparison between collagen and other groups, ^#^comparison between the MHMs/collagen and S-MHMs/collagen groups, *p* < 0.05).

Reconstructed micro-CT images show that a greater amount of new bone formed in the defects implanted with the S-MHMs/collagen scaffolds than in the defects implanted with the MHMs/collagen or collagen scaffolds (Fig. 9A). Greater BV/TV and BMD were observed in the S-MHMs/collagen group (20.70 ± 6.00% and 394.67 ± 72.45 mg cm^−3^) compared with those in the MHMs/collagen group (10.43 ± 4.33% and 172.67 ± 39.11 mg cm^−3^) and the collagen group (2.57 ± 0.89% and 55.67 ± 13.32 mg cm^−3^) (Fig. 9B,C).

Furthermore, neovascularization in the defects was determined by microfil perfusion and micro-CT imaging. As shown in Fig. 9D, there were more newly formed blood vessels in the defects implanted with the S-MHMs/collagen scaffolds compared with those implanted with the MHMs/collagen or collagen scaffolds. The blood vessel area and number in the S-MHMs/collagen group (19.62 ± 1.44% and 54 ± 9) were greater than those in the MHMs/collagen group (10.49 ± 1.63% and 28 ± 3) and the collagen group (2.44 ± 0.65% and 4 ± 2) (Fig. 9E,F).

### Histological evaluation

**Figure 10:**
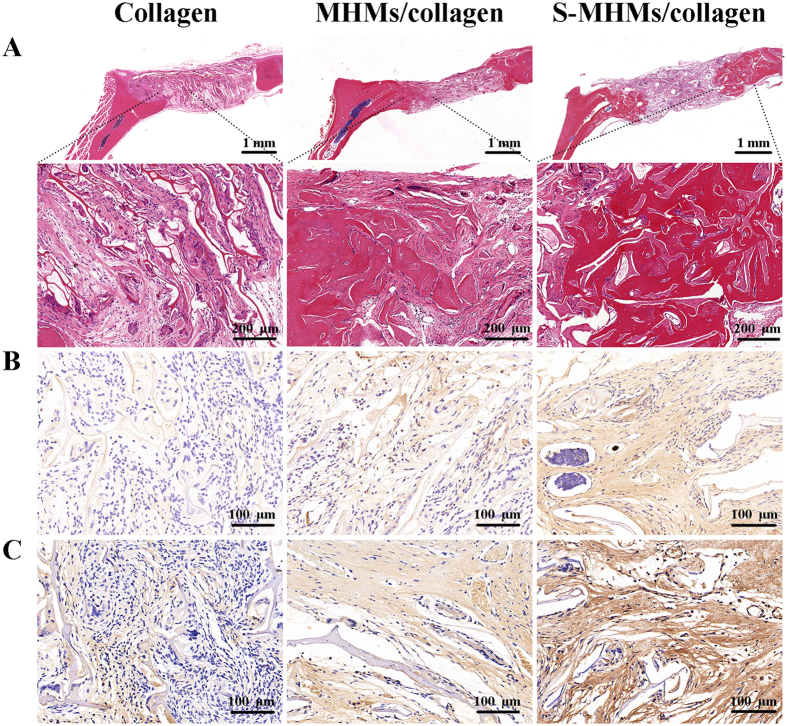
Histological assessment of bone regeneration in each group. (**A**) HE staining; (**B**,**C**) IHC staining for OCN and OPN.

HE staining of representative sections from each group is shown in Fig. 10A. In the collagen group, the defect area was mainly occupied with connective tissue, and typical bone tissue was scarcely observed. The newly formed bone in the defects filled with S-MHMs/collagen scaffolds was greater than in those implanted with MHMs/collagen scaffolds.

The IHC staining results are shown in Fig. 10B and C. The osteogenic markers, including OCN and OPN, were highly expressed in the S-MHMs/collagen group. In comparison, there was limited positive staining for OCN or OPN in the defects implanted with MHMs/collagen or collagen scaffolds.

**Figure 11:**
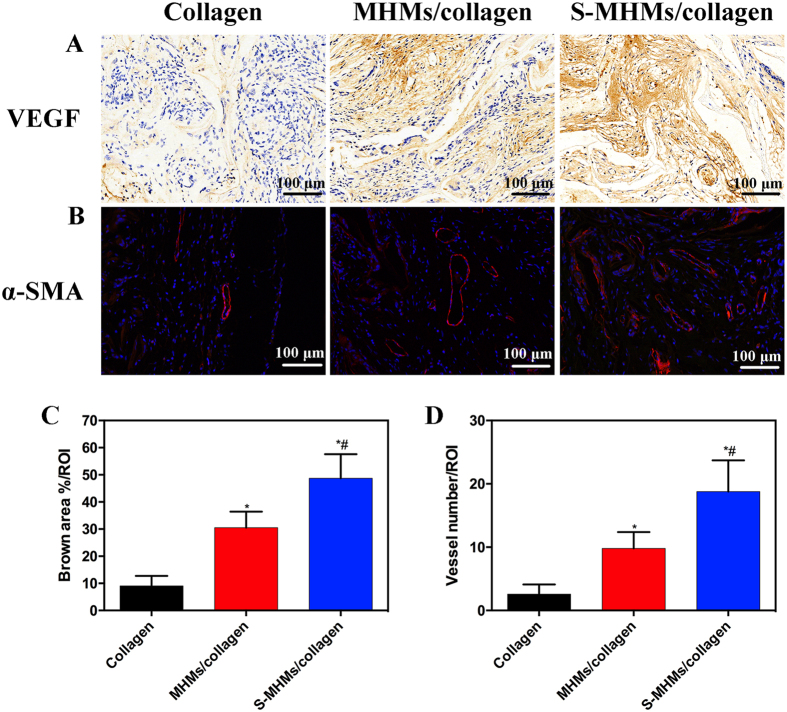
Histological assessment of neovascularization in each group. (**A**) IHC staining for VEGF; (**B**) IF staining for α-SMA (red); (**C**) Quantification of the brown area surface to the total surface of images; (**D**) Quantification of the newly formed blood vessels (red fuorescence). (*Comparison between collagen and other groups, ^#^comparison between the MHMs/collagen and S-MHMs/collagen groups, *p* < 0.05).

Similarly, the expression of VEGF was more intensive in the S-MHMs/collagen group than that in the MHMs/collagen or collagen group (Fig. 11A and C). IF staining for the α-SMA (red) showed that the number of blood vessels in the defects implanted with S-MHMs/collagen scaffolds was higher than that in the MHMs/collagen or collagen group (Fig. 11B and D).

## Discussion

In the present study, water-insoluble simvastatin was successfully incorporated into the MHMs with high specific surface area and hierarchical nanostructure. *In vitro*, the S-MHMs not only enhanced the osteogenic differentiation of rBMSCs but also promoted the angiogenesis in EA.hy926 cells. Furthermore, the S-MHMs were homogenously incorporated into the collagen matrix to construct a pleiotropic bone tissue-engineering scaffold, which showed a sustained release of simvastatin. The *in vivo* results demonstrated that the S-MHMs/collagen scaffolds enhanced the bone regeneration and neovascularization simultaneously in critical-sized rat calvarial defects 8 weeks post-implantation.

The concentrations of S-MHMs used for the *in vitro* experiment were determined according to the loading efficiency of simvastatin. The 0.6, 6, and 60 μg/mL S-MHMs corresponded to approximately 0.1, 1 and 10 μM of simvastatin, respectively. Firstly, we examined the cytotoxicity of the MHMs and S-MHMs and found that MHMs and S-MHMs at concentrations of 0.6 and 6 μg/mL showed no appreciable cytotoxicity, whereas MHMs and S-MHMs at the concentration of 60 μg/mL inhibited the cell proliferation, which may be associated with the adverse effects resulting from high concentrations of simvastatin and microspheres. It has been reported that simvastatin at the concentration of 10 μM inhibited the proliferation of HUVECs^35^. Then, we tested the ALP activity, a common marker in osteogenesis research, and found that the rBMSCs treated with S-MHMs expressed higher ALP activity than those treated with MHMs. The osteogenic activity of S-MHMs was further verified by RT-qPCR. The expression levels of osteogenesis-related genes, including Runx2, ALP, BMP-2, OCN and OPN, were apparently up-regulated by S-MHMs after 7 and 14 days of culture. These results demonstrated that the loaded simvastatin maintained its pharmacological activities. It is likely that the simvastatin released from the S-MHMs acts effectively on the rBMSCs to induce osteogenic differentiation. It was reported that statins could promote osteoblast differentiation by increasing BMP-2 expression, which might be mediated via the activation of Akt and MAPK signaling pathways^36^. The BMP-2 binds to a specific receptor (receptor II) at the cell membrane and forms a complex that phosphates Smad protein^37^. The phosphorylated Smad complex translocates to the nucleus and regulates the transcriptional activity of the target genes^38^. BMP-2-induced osteogenesis is regulated by Runx2, which was also up-regulated in the rBMSCs cultured with the S-MHMs. The molecular mechanism of the simvastatin-induced osteogenesis is beyond the scope of this study and remains further investigation.

In this study, the pro-angiogenic activity of the S-MHMs was assessed using cell migration and tube formation assays. The results showed that the S-MHMs apparently enhanced the migration and tube formation of the EA.hy926 cells in a concentration-dependent manner, indicating the simvastatin released from S-MHMs stimulated the angiogenic differentiation of the EA.hy926 cells. Previous studies have demonstrated that the angiogenesis process of vascular endothelial cells induced by simvastatin was mainly mediated by the PI3-kinase/Akt and Erk signaling pathways^35^,^39^,^40^. Angiogenesis is a process orchestrated by multiple angiogenic factors, among which VEGF is an essential growth factor regulating the critical steps of angiogenic process^41^. VEGF exerts its effects on endothelial cells through binding to the tyrosine kinase receptors of VEGFR-1, 2 and 3 and induces the angiogenesis process including the regulation of endothelial cell migration^41^,^42^. It was reported that simvastatin promoted skin wound healing by enhancing the expression of VEGF in an experimental model of diabetes^43^. Consistent with this study, the S-MHMs improved the expression of VEGF in a concentration-dependent manner. Furthermore, the expression of HIF-1α in rBMSCs cultured with S-MHMs was examined. As a result, the S-MHMs remarkably enhanced the expression of HIF-1α in a concentration-dependent manner. HIF-1 is a transcription factor composed of 2 subunits, HIF-1α and HIF-1β, and HIF-1α acts as a cellular oxygen sensor that induces a response to low oxygen tension. Activation of the HIF pathway stimulates the transcription of multiple hypoxia response genes, among which VEGF is a major target^44^. It was reported that simvastatin could protect HIF-1α from degradation by inhibiting prolyl-4-hydroxylase 3 (PHD-3), which hydroxylates the two proline residues in the oxygen-dependent degradation domain of HIF-1α under normoxic conditions and leads to interactions with the von Hippel-Lindau (VHL) ubiquitin ligase complex that promotes ubiquitin-mediated proteolysis of the HIF-1α subunit^45^.

Scaffolds derived from type I collagen fabricated through a freeze-drying process showed immense potential in bone tissue engineering^46^,^47^,^48^. Calcium phosphate-incorporated collagen scaffolds have been investigated as a means to improve the bioactivity and mechanical properties, mimicking the extracellular matrix of bone^49^,^50^. Herein, a novel S-MHMs/collagen composite scaffold was constructed by incorporating the S-MHMs into the collagen matrix. With the aid of MHMs, simvastatin was homogenously incorporated into the hydrophilic collagen matrix. Encouragingly, the S-MHMs/collagen scaffold showed a sustained release of simvastatin for up to 3 weeks, whereas the S-MHMs showed a burst release and trended toward an equilibrium release for 60 h. The drug release process of S-MHMs/collagen scaffold was probably governed by the release kinetics of S-MHMs system and the diffusion process within collagen matrix. The combination of collagen greatly slowed down the drug release rate and prolonged the total drug release time. It is assumed that the collagen may act as a buffering system to curb the burst release of simvastatin as a result of the interaction between the simvastatin molecules and collagen matrix. Delivery of simvastatin for a long period is desirable to exert its stimulatory effects during different phases of bone regeneration including the inflammation, proliferation and remodeling stages^15^. Because high concentrations of simvastatin may cause cell toxicity, controlled release of simvastatin from the S-MHMs/collagen scaffolds is therefore desirable at concentrations pertinent to induce osteogenesis and angiogenesis.

Having demonstrated that the simvastatin was released in controlled profile from the S-MHMs/collagen scaffold, we investigated whether the scaffolds were capable of eliciting pro-osteogenic and pro-angiogenic responses *in vivo*. Eight weeks post-implantation, the results demonstrated that the bone defects implanted with the S-MHMs/collagen scaffolds achieved superior bone regeneration and neovascularization than those implanted with the MHMs/collagen or collagen scaffolds. The simvastatin released from the S-MHMs/collagen scaffolds within the bone defects may encourage new bone formation by coupling the osteogenesis and angiogesis processes. It is assumed that the simvastatin released locally was proportional to the degradation of the scaffolds and acted effectively on stem cells present in the surrounding tissue to induce osteogenesis. The histomorphometry analysis demonstrated that the degradation of scaffolds was apparent and scaffolds remnants were scarcely present within the defects after 8 weeks of implantation. The biodegradation of the scaffolds may accelerate the release of simvastatin compared with the *in vitro* release condition. In addition to the osteoinductive effects on local MSCs, the simvastatin may also recruit circulating or surrounding MSCs to the implantation site. Liu *et al*. reported that simvastatin could enhance the migration of BMSCs and promote bone regeneration in mouse critical-sized calvarial defects^51^. Consistent with this study, our *in vitro* results showed that the S-MHMs enhanced the migration of rBMSCs in a concentration-dependent manner. The interplay between osteogenesis and angiogenesis leads to better bone healing^52^,^53^. It is well recognized that sufficient vascularization plays an essential role during bone reconstruction^54^. The S-MHMs/collagen scaffolds effectively enhanced the vascular response in the critical-sized bone defects by micro-CT and histological assessments. Moreover, consistent with the *in vitro* results, the expression of VEGF was also enhanced in the S-MHMs/collagen group. Exogenous administration of VEGF has been shown to enhance bone formation in both rabbit and mouse models^55^,^56^. Thus, the cross-talk of the pleiotropic effects of S-MHMs/collagen scaffolds, including pro-osteogenic effect, pro-angiogenic effect and BMSCs recruitment, may synergistically contribute to the superior bone regeneration.

## Conclusion

In the present study, water-insoluble simvastatin was successfully incorporated into MHMs with high specific surface area, and the S-MHMs showed osteogenic and angiogenic activities simultaneously. Furthermore, a novel S-MHMs/collagen composite scaffold was constructed by incorporating the S-MHMs into the collagen matrix. With the aid of MHMs, simvastatin was homogenously incorporated into the hydrophilic collagen matrix. The S-MHMs/collagen scaffold showed a sustained release of simvastatin for 21 days. More importantly, the S-MHMs/collagen scaffold enhanced the bone regeneration accompanied with improved neovascularization. These findings are considered to promise the potential application of S-MHMs/collagen scaffold as a therapeutically relevant biomatrix platform for bone regeneration by enhancing osteogenesis and angiogenesis simultaneously.

## Materials and Methods

### Synthesis and characterization of the MHMs

In a typical experiment^57^, 0.122 g of fructose 1,6-bisphosphate trisodium salt (FBP) (Sangon Biotech, China) was dissolved in 15 mL of deionized water, and then the above solution was added dropwise to 25 mL CaCl_2_ aqueous solution (CaCl_2_, 0.111 g, Sinopharm) under magnetic stirring at room temperature. The pH value of the mixed solution was maintained at 10 by dripping 1 M NaOH aqueous solution. After continuous stirring for 10 min, the resulting solution was transferred into a 60 mL autoclave, and heated in a microwave oven (MDS-6, Sineo, China) to 140 °C for 10 min. After cooling to room temperature, the product was separated by centrifugation, washed with deionized water, and dried at 60 °C for 24 h.

The morphology of the MHMs was observed using a field-emission scanning electron microscope (FE-SEM, SU8200, Japan) and a transmission electron microscope (TEM, Hitachi H-800, Japan). The X-ray diffraction (XRD) pattern of the MHMs was recorded using an X-ray diffractometer (Rigaku D/max 2550 V, Cu_Kα_ radiation, λ = 1.54178 Å). The dynamic light scattering (DLS) measurement was taken on a zeta potential analyzer (ZetaPlus, Brookhaven Instruments Corporation). The Brunauer-Emmett-Teller (BET) specific surface area and pore size distribution were measured by a specific surface area and pore size analyzer (V-sorb 2800 P, Gold APP, China).

### *In vitro* simvastatin loading and release.

The simvastatin (Sigma, USA) loading experiment was performed as follows. The MHMs (100 mg) were dispersed into an ethanol solution of simvastatin (5.0 mg mL^−1^, 20 mL). Then, the suspension was shaken in a sealed vessel at a constant rate (120 rpm) at 37 °C for 24 h. The suspension was centrifuged and freeze-dried at −20 °C for 24 h to obtain the simvastatin-loaded MHMs (S-MHMs). Fourier transformed infrared (FTIR) spectra were obtained on an FTIR spectrometer (FTIR-7600, Lambda Scientific, Australia). The concentration of simvastatin in the ethanol solution before and after loading was measured by a UV-vis spectrophotometer (UV-2300, Techcomp) at a wavelength of 238 nm to determine the drug-loading efficiency of simvastatin. The drug-loading efficiency was calculated using the following equation:


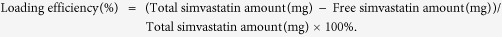


For the simvastatin release assay, 10 mg of S-MHMs was immersed into 15 mL of normal saline (NS) at 37 °C with constant shaking (120 rpm). At given time intervals, 0.5 mL of drug-release medium was extracted and measured by UV/Vis absorption spectroscopy at a wavelength of 238 nm and replaced with the same volume of fresh NS.

### Culture of rBMSCs and EA.hy926 cells

All experimental protocols were approved by the Animal Care and Experiment Committee of Sixth People’s Hospital affiliated to School of Medicine, Shanghai Jiao Tong University. The rBMSCs were obtained from the femur and tibia of 4-week-old Sprague-Dawley (SD) rats. Briefly, the marrow of the midshaft femur and tibia was flushed out and suspended in complete medium (CM, α-MEM (Gibco, USA) supplemented with 10% fetal bovine serum (FBS, Gibco, USA) and 1% (v/v) penicillin/streptomycin (Gibco, USA)). Non-adherent cells were removed after 48 hours. The rBMSCs from passage 2 were used for the following experiment.

The human endothelial cell line EA.hy926 is an immortalized hybridoma line that retains features of endothelial cells in culture, including a cobblestone appearance with formation of capillary-like tubes. Cells were cultured at 37 °C in humidified air containing 5% CO_2_.

### Cell proliferation assay

The Cell Counting Kit-8 assay (CCK- 8; Dojindo Molecular Technologies, Inc., Japan) was used to determine the metabolic activity of rBMSCs and EA.hy926 cells. Briefly, the rBMSCs or EA.hy926 cells were seeded in a 96-well plate at a density of 3 × 10^3^ cells/well and cultured with different concentrations of MHMs or S-MHMs (0.6, 6 or 60 μg/mL). At days 1, 3 and 7, the culture medium was removed and 100 μL of fresh medium with 10% CCK-8 solution was added to each well. After incubation for 4 h, aliquots (100 μL) from each well were transferred to a new 96-well plate for measurement. The absorbance was measured with a microplate reader (Bio-Rad 680, USA) at a wavelength of 450 nm.

### Alkaline phosphatase (ALP) activity and staining

To evaluate the biological activity of MHMs and S-MHMs, a bioassay of the *in vitro* cell culture was carried out^58^. Briefly, rBMSCs were seeded into a 24-well plate at a density of 5 × 10^4^ cells/well and cultured with different concentrations of MHMs or S-MHMs (0.6, 6 or 60 μg/ml). ALP activity was first assessed quantitatively using an ALP Detection Kit (Jiancheng Technology, Nanjing, China) on day10. The ALP activity was measured according to the manufacturer’s instructions and normalized to the total protein content determined by the BCA method. For ALP staining, the monolayer cells were rinsed with phosphate buffered saline (PBS) three times and fixed with 4% paraformaldehyde solution for 10 min. Then, the cells were stained with fast 5-bromo-4-chloro-3-indolylphosphate and nitroblue tetrazolium (BCIP/NBT) ALP substrate (Beyotime Biotechnology, China) for 30 min at room temperature. The reaction was terminated by removing the substrate solution and washing with distilled water.

### Real-time quantitative reverse transcription PCR (RT-qPCR) analysis

The effects of MHMs and S-MHMs on the osteogenic differentiation of rBMSCs were assessed by measuring the mRNA expression of runt-related transcription factor 2 (Runx2), bone morphogenic protein 2 (BMP-2), ALP, osteocalcin (OCN), and osteopontin (OPN). Total RNA was isolated using the Trizol reagent (Invitrogen, USA) and 500 ng RNA was reversed transcribed into complementary DNA (cDNA) using a PrimeScript 1st Strand cDNA Synthesis Kit (Takara, Japan) according to the manufacturer’s instructions. Expression was quantified by the ABI Prism 7900 Thermal Cycler (Applied Biosystem, Australia) using a real-time PCR kit (SYBR Premix EX Taq, Takara, Japan). The gene expression levels were normalized to that of the housekeeping gene GAPDH and relative gene expression was analyzed with the 2^−ΔΔCt^ method.

### Cell migration and tube formation assays

The effects of the S-MHMs on the migration of EA.hy926 cells and rBMSCs were evaluated with a transwell assay. Briefly, 5 × 10^4^ cells were seeded in the upper chamber of a 24-well plate (Corning; pore size = 8 μm). Then, 500 μL of medium with different concentrations of MHMs or S-MHMs was added to the lower chamber. After incubation for 24 h, the filter was gently removed, and the cells on the upper surface of the membrane were removed with a cotton swab. Cells on the lower surface of the membrane were fixed with 4% paraformaldehyde and stained with 0.5% crystal violet for half an hour. The cells that migrated to the lower chamber were observed with an optical microscope. Finally, the crystal violet on the membrane was solubilized with 500 μL of 33% acetic acid solution. The optical density (OD) of the plates was measured at 595 nm wavelength using a microplate reader.

EA.hy926 cells were used in the tube formation assay. Briefly, 50 μL of growth factor-depleted Matrigel (Becton Dickinson, MA) was added to a 96-well plate and allowed to gel for half an hour at 37 °C. Then, the Matrigel was overlaid with a suspension of cells (2 × 10^4^ cells/well) that have been pretreated with different concentrations of MHMs or S-MHMs (0.6, 6 or 60 μg/ml) for 30 min. After incubation for 8 h, the tube formation activity was estimated by counting the number of complete capillaries connecting individual points of the polygonal structures in an optical microscope.

### Western blot assay for HIF-1α and VEGF

Briefly, rBMSCs were seeded in 6-well plates at a density of 1 × 10^5^ cells/well and cultured with different concentrations of MHMs or S-MHMs for 48 h. For detection of HIF-1α, CoCl_2_ was added to the medium at a final concentration of 100 μM. Equal amount of protein from each sample was separated on SDS-PAGE gels and then transferred to polyvinylidenedifluoride membranes (Millipore, MA, USA). After being blocked with milk for 3 h, the membranes were incubated with primary antibodies against HIF-1α (1:1000, rabbit anti rat; CST, USA), VEGF (1:1000, rabbit anti rat; Abcam, Australia) or β-actin (1:5000, goat anti rabbit; Abcam, Australia) overnight at 4 °C. Finally, the membranes were visualized with horseradish peroxidase (HRP)-conjugated goat anti-rabbit (Beyotime, China) using the ECL plus reagents (Solarbio, China) under a UVItec ALLIANCE 4.7 gel imaging system.

### Scaffold fabrication

Three different types of scaffolds corresponding to three groups were prepared: (1) collagen scaffold, (2) MHMs/collagen scaffold, and (3) S-MHMS/collagen scaffold. Briefly, the MHMs or S-MHMs were added to the collagen suspension (40 mg g^−1^) under vigorous stirring, and the weight ratio between collagen and MHMs or S-MHMs was 1:1. Then, the homogeneous mixture was loaded into a 48-well plate, frozen at −20 °C for 24 h and lyophilized at −20 °C for 48 h. The collagen matrix of the lyophilized scaffolds was cross-linked in a solution containing 20 mM N-(3-dimethylaminopropyl)-N’-ethylcarbodiimide hydrochloride (EDC, Sigma–Aldrich) and 8 mM N-hydroxysuccimide (NHS, Sigma–Aldrich) in 80/20 ethanol/deionized water for 2 h^59^. The cross-linked scaffolds were subsequently rinsed three times with deionized water and dried at 37 °C for further use. The morphology of the scaffolds was observed using SEM.

The water-absorbing capacity of the scaffolds was measured according to a previous method^60^. Briefly, the dried scaffolds were cut into Φ10 × 5 mm cylinders and weighed (W_1_). Then, the scaffolds were immersed in deionized water for 12 h and weighed (W_2_) after removing the surface excess water by filter papers. The hydroscopicity was calculated as follows:


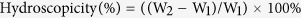


### Release test of the S-MHMs/collagen scaffold

Briefly, 25 mg of S-MHMs/collagen scaffold was immersed in 15 mL of NS at 37 °C under constant shaking (120 rpm). At given time intervals, 0.5 mL of release medium was collected and replaced with fresh NS, the concentration of released simvastatin was measured by a UV-vis spectrophotometer at a wavelength of 238 nm.

### Cell attachment and live/dead assay of the rBMSCs on scaffolds

Briefly, the rBMSCs were seeded on the scaffolds (Φ10 mm × 1 mm) at a density of 1 × 10^5^ cells/scaffold and cultured for 3 days. For SEM observation, the scaffolds with cells were fixed in 2.5% glutaraldehyde solution for 4 h and dehydrated in a graded ethanol series. After freeze-drying, the scaffolds were coated with platinum and observed using SEM.

For cell live/dead staining, a live/dead viability cytotoxicity kit (Invitrogen) was used according to the manufacturer’s instructions. For cytoskeleton staining, the scaffolds were fixed in 4% paraformaldehyde for 30 min, rinsed with PBS three times and then pretreated with 0.5% triton for 5 min. Finally, rhodamine-phalloidin (Sigma, USA) and DAPI (Beyotime, China) were prepared and incubated with the scaffolds for 30 min and 5 min, respectively. The cells on the scaffolds were observed with a confocal laser scanning microscope (CLSM, Zeiss, LSM 510).

### Animal surgical procedures

All surgical procedures were performed on 12-week-old male SD rats. Briefly, a 1.0–1.5 cm sagittal incision was made on the scalp, followed by blunt dissection of periosteum. Two defects 5 mm in diameter were created in each rat by an electric trephine (Nouvag AG, Goldach, Switzerland). Then, the calvarial defects were filled with the scaffolds (Φ 5 mm × 2 mm). Finally, the incision was closed by suturing the periosteum and skin separately. Twenty-four rats were randomly allocated into the following study groups: (1) collagen scaffold (n = 8), (2) MHMs/collagen scaffold (n = 8) and (3) S-MHMs/collagen scaffold (n = 8).

### Microfil perfusion

For *in vivo* evaluation of neovascularization, the rats were perfused with Microfil (Microfil MV-122, Flow Tech, Carver, MA) after they were euthanized at 8 weeks post-surgery^61^. Briefly, following a midline thoracotomy, the left ventricle was penetrated with an angiocatheter, and the right auricle was incised. Then, 50 mL of heparinized NS and 10 mL of Microfil working solution were successively perfused at 2 mL min^−1^. Finally, the specimens were set at 4 °C overnight to ensure complete polymerization of the contrast agent.

### Micro-CT assay

To evaluate calvarial defect repair and blood vessel formation, micro-CT (Skyscan 1176, Kontich, Belgium) scan was performed at a resolution of 18 and 9 μm for undecalcified and decalcified samples, respectively. The 3-D images were reconstructed by CTVox program (Skyscan Company). Bone volume to total volume (BV/TV), local bone mineral density (BMD), blood vessel area and number in the bone defects were determined using CTAn program (Skyscan Company). The volume of interesting (VOI) was defined as the whole calvarial bone defect. A threshold of 120 in a scale from 0 to 255 was adopted to affirm the bone tissues.

### Histological assessment for bone formation and neovascularization

The decalcified specimens were dehydrated and subsequently embedded in paraffin. Sections (5 μm thickness) were stained with hematoxylin and eosin (HE). The immunohistochemical (IHC) staining for OCN, OPN and VEGF was performed to evaluate the osteogenic and angiogenic activities of the scaffolds. Furthermore, immunofluorescence (IF) staining for alpha-smooth actin was performed to identify the newly formed blood vessels. Image J software was used to calculate the percentage of brown area to the total area of the images, and the number of blood vessels were manually calculated. At least 5 images from each group were analyzed.

### Statistical analysis

The means and standard deviations of data were calculated. One-way analysis of variance and Student-Newman-Keuls post hoc tests were used to determine the level of significance, with *p* < 0.05 being considered statistically significant.

### Ethics statement

All experimental protocols were approved by the Animal Care and Experiment Committee of Sixth People’s Hospital affiliated to School of Medicine, Shanghai Jiao Tong University, and all procedures were carried out in accordance with the approved guidelines.

## Additional Information

**How to cite this article:** Yu, W.-L. *et al*. Enhanced osteogenesis and angiogenesis by mesoporous hydroxyapatite microspheres-derived simvastatin sustained release system for superior bone regeneration. *Sci. Rep.* **7**, 44129; doi: 10.1038/srep44129 (2017).

**Publisher's note:** Springer Nature remains neutral with regard to jurisdictional claims in published maps and institutional affiliations.

## Supplementary Material

Supplementary Information
