# Supplementary material for: Enhanced osteogenesis and angiogenesis by mesoporous hydroxyapatite microspheres-derived simvastatin sustained release system for superior bone regeneration
Source: Sci Rep. 2017 Mar 13;7:44129. doi: 10.1038/srep44129 (PMC5347005; doi:10.1038/srep44129)
Supplement: Supplementary Information [file srep44129-s1.pdf]

Supplementary Information

Enhanced osteogenesis and angiogenesis by mesoporous hydroxyapatite microspheres-derived simvastatin sustained release system for superior bone regeneration

Wei-Lin Yu [1 *], Tuan-Wei Sun [2, 3 *], Chao Qi [2, 3], Hua-Kun Zhao [1], Zhen-Yu Ding [1], Zhi-Wang Zhang [1], Ben-Ben Sun [1], Ji Shen [1], Feng Chen [2, 3], Ying-Jie Zhu [2, 3], Dao-Yun Chen [1]，Yao-Hua He [1, 4]

[1]Department of Orthopedics, Shanghai Jiao Tong University Affiliated Sixth People's Hospital, 600 Yishan Road, Shanghai 200233, China.

[2]State Key Laboratory of High Performance Ceramics and Superfine Microstructure，Shanghai Institute of Ceramics, Chinese Academy of Sciences, 1295 Dingxi Road, Shanghai 200050, China.

[3]University of Chinese Academy of Sciences, 19 Yuquan Road, Beijing 100049, China.

[4]Shanghai Jiao Tong University Affiliated Sixth People's Hospital, School of Biomedical Engineering, 600 Yishan Road, Shanghai 200233, China.

Corresponding Authors

Y.-H.H. (email: hyhua18930177339@163.com)

D.-Y.C (email: chendaoyun324309@163.com)

Y.-J.Z. (email: y.j.zhu@mail.sic.ac.cn)

Supplementary Figure S1

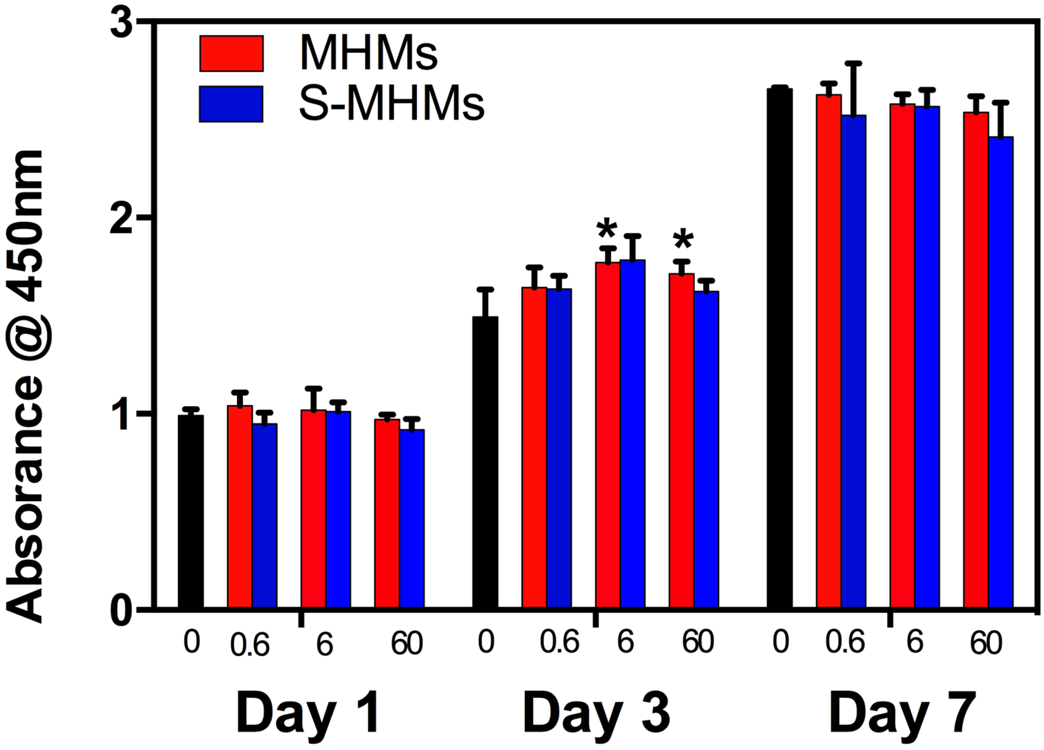

Figure S1. The effects of MHMs and S-MHMs on the proliferation of EA.hy926 cells. ‘‘0, 0.6, 6 and 60’’ represent the concentrations of MHMs and S-MHMs (μg/mL). (*Comparison between MHMs group and the blank control, $p<0.05$).
